# Effects of Holder Pasteurization on 15-F2t-Isoprostane and Total Antioxidant Power in Donor Human Milk

**DOI:** 10.3390/biom16030437

**Published:** 2026-03-13

**Authors:** Valeria Bellisario, Samar El Sherbiny, Giulia Squillacioti, Alessia Spadavecchia, Elisabetta Punziano, Alessandra Coscia, Chiara Peila, Roberto Bono

**Affiliations:** 1 Department of Public Health and Pediatrics, University of Turin, 10126 Turin, Italy; valeria.bellisario@unito.it (V.B.); samar.elsherbiny@unito.it (S.E.S.); giulia.squillacioti@unito.it (G.S.); alessandra.coscia@unito.it (A.C.); roberto.bono@unito.it (R.B.); 2 Neonatal Unit, Department of Public Health and Pediatric Sciences, University of Turin, 10126 Turin, Italy; alessia.spadavecchia@unito.it (A.S.); elisabetta.punziano@unito.it (E.P.); 3 Neonatal Care Unit of the University, City of Health and Science Hospital of Turin, 10100 Turin, Italy; 4 Research Center for Training, Health Education and Local Empowerment, University of Turin, 10126 Turin, Italy

**Keywords:** Holder pasteurization, human donor milk, 15-F2t-isoprostane, oxidative stress

## Abstract

Human milk is the optimal standard for neonatal nutrition, particularly for preterm infants. Several conditions associated with oxidative stress (OS) may be transmitted from mother to infant through milk, making the preservation of milk quality essential. When maternal milk is unavailable, donor human milk (DM) is commonly used and treated with Holder pasteurization (HoP) to ensure microbiological safety, although this process may affect bioactive components. This study aimed to evaluate the impact of HoP on OS biomarkers, specifically total antioxidant power (TAP) and 15-F2t-isoprostane, using colorimetric and ELISA methods as cost-effective alternatives to analytical gold standards. Twenty paired DM and HoP samples from the Human Milk Bank of Sant’Anna Hospital (Turin, Italy) were analyzed. No significant differences were observed in TAP levels between DM and HoP samples. In contrast, 15-F2t-isoprostane concentrations were significantly lower in DM compared to pasteurized milk (3.16 (1.59–5.27) vs. 0.76 (0.62–1.54), *p*-value < 0.001). This reduction remained consistent after stratification by sampling day. These findings suggest that HoP may reduce oxidative stress markers in donor milk, potentially limiting neonatal exposure to maternal oxidative imbalance. Although this effect could offer protective benefits for vulnerable preterm infants, further studies are needed to clarify the clinical implications of HoP on redox status and neonatal outcomes.

## 1. Introduction

Human milk is currently recommended as the gold standard for newborn nutrition due to the several bioactive and immunomodulatory factors contained in it [1,2,3,4]. However, whenever own mothers’milk (OMM) is not available, donor milk (DM) is the optimal alternative, particularly for preterm infants [1,2,3]. To ensure safety before its administration, pasteurization of DM is indicated to inactivate potentially dangerous infective agents like HIV, HBV, cytomegalovirus or bacterial infections like *Escherichia coli*, *Staphylococcus aureus*, and *Streptococci* [5,6]. The currently recommended pasteurization method by worldwide guidelines of Human Milk Banks is Holder pasteurization (HoP), in which a phase of rapid heating, up to 62.5 °C for 30 min, is followed by a phase of cooling at room temperature and then by a phase of rapid cooling at 4 °C [6,7,8]. Thanks to HoP, many beneficial and protective effects of human milk are retained, starting from ensuring the necessary protection from pathogenic microorganisms. This practice can, however, affect human milk in some nutritional and biological aspects [9], like reductions in energy content [10], immunoglobulin concentration, especially IgA, IgM, and IgG [11,12,13,14], lactoferrin, lysozyme [15,16,17], enzymes concentration [18,19], and water-soluble vitamins concentration [20].

On the other hand, other evidence shows that HoP does not affect the concentration of protein and amino acids [18,21,22,23], cytokines [24,25,26], growth factors (except for HGF and IGF 1-2) [27], fat-soluble vitamins [28], lipids [18,24,26,29,30,31], saccharides [10,11,14,23,32,33,34] and organic acids [35]. A systematic review by Peila [36] highlighted that findings regarding the effects of HoP on human milk are neither comprehensive nor conclusive, indicating the need for further studies to validate the real impact of this process on human milk. Regarding the effect that pasteurization can have on oxidative stress (OS), some data are available about total antioxidant power (TAP), but no data are available about the impact of HoP on 15-F2t-isoprostane (15-F2t-Isop) concentration. TAP is a marker able to provide a measure of the ability of our body to counteract reactive oxygen species [37]. 15-F2t-Isop is a prostaglandin-like compound originating from the peroxidation of arachidonic acid and considered one of the most reliable markers of OS in vivo [38]; it is already employed in many different biological fluids [39,40,41,42,43]. Overall, OS markers and the oxidative balance in DM during processing remain insufficiently characterized [44,45]. OS is a crucial factor to address when considering DM administration, particularly for preterm infants [46,47]. Given their limited antioxidant capacity, the established cause-and-effect relationship between several prematurity-related disorders, and an imbalance between OS and antioxidative defenses, further investigation into these parameters is essential [48,49].

Hence, the present study was designed to test the hypothesis that Holder pasteurization modifies the redox balance of donor human milk by reducing oxidative damage markers while preserving overall antioxidant capacity. Specifically, total antioxidant power (TAP) and 15-F2t-isoprostane were quantified and compared in pasteurized and non-pasteurized donor milk to evaluate the impact of thermal processing on oxidative stress status. In parallel, the applicability of colorimetric assays and enzyme-linked immunosorbent assays (ELISAs) was assessed to determine if they were practical and cost-effective alternatives to analytical gold standard methods, such as liquid chromatography–mass spectrometry (LC-MS) for 15-F2t-isoprostane and ferric reducing antioxidant power (FRAP) for TAP. This combined biological and methodological approach aimed to provide a more comprehensive evaluation of redox homeostasis in human milk and to facilitate larger-scale studies in this field.

## 2. Materials and Methods

### 2.1. Sample Collection

Breast milk samples from six women were collected from the donor human milk stored at the Human Milk Bank (HMB) of Sant’Anna Hospital (A.O.U. Città della Salute e della Scienza, Turin, Italy), during a sampling campaign in May 2024.

Milk samples were collected from mothers who met the following inclusion criteria: good health status, full-term pregnancy, and no history of pathological conditions. These criteria were chosen to minimize potential influences on the inflammatory profile. The HMB provided these samples to the researchers in response to a formal request, and only after donors provided informed consent directly to the HMB during the recruitment and donation process. As these HMB-supplied samples were specifically for this study and contained no donor information, no additional informed consent or ethical approval was required. A sample of fresh milk was collected twice from each donor, once on the second day and once on the third day after delivery, for a total of 24 samples. Milk, as usual, was extracted using an electric breast pump into high-density sterile polyethene sealed bottles. Each sample underwent two treatments. Half was pasteurized according to the HoP method and the other half was not. From each container, 10 mL of human milk was aliquoted and divided into two fractions: the first was stored unaltered at +4 °C; the second was pasteurized at the HMB and then stored at +4 °C. HoP pasteurization was performed at the HMB of Sant’Anna Hospital with a Sterifeed Pasteuriser by Medicare Colgate Ltd. (Cullompton, UK): milk was heated to 62.5 °C for 30 min, followed by a rapid and controlled cooling of milk samples to 10 °C within approximately 20 min through immersion in cold water. All samples were transported to the laboratory within 2 h of pasteurization, where they were aliquoted and stored at −80 °C until analysis.

### 2.2. Sample Preparation and Analysis

Milk samples were obtained from the milk biobank of Sant’Anna Hospital in aliquots of 2 mL for each sample. These 15-F2t-Isop concentrations were measured through the competitive ELISA Kit (EA84) from Oxford Biomedical Research (Rochester Hills, MI, USA). As milk often contains high concentrations of proteins or other substances that could interfere with the immunoassay, purification through solid phase extraction (SPE) was required before use in this assay. For the SPE phase, Sep-Pak C-18 Cartridge and Sep-Pak Silica Cartridge (500 mg Sorbent per Cartridge, 55–105 µm and 500 mg Sorbent per Cartridge, 55–105 µm, respectively) were purchased from Waters (Milford, MA, USA), while ethyl acetate, methanol, and heptane were bought from Sigma Aldrich (Milano, Italy) and nitrogen was provided by SIAD S.p.A (Bergamo, Italy). Before the purification process, samples were brought to a pH of 3. A C-18 cartridge was pre-washed with 5 mL of ethanol, followed by 5 mL of dH_2_O, pH 3 (the cartridge was prevented from becoming dry), and samples were loaded using a flow rate of 1 mL/min. After that, the column was washed with 10 mL of dH_2_O, pH 3, and 10 mL of heptane. At this point, the waste was discarded, and Falcon tubes were used to collect the samples. Elution was performed using 10 mL of an ethyl acetate and heptane solution. Eluted samples were removed from the manifold and set aside for the subsequent phase of extraction. SPE SILICA columns were placed in the manifold and pre-washed with 5 mL of MeOH and 5 mL of ethyl acetate. Samples collected from the C-18 column were loaded with a flow rate of 1 mL/min and then washed with 5 mL of MeOH and 5 mL of ethyl acetate. Once the waste was discarded, new Falcon tubes were placed in the manifold to perform the final elution step with 5 mL of ethyl acetate and MeOH solution. Samples were dried with nitrogen and stored at −80 °C. As previously mentioned, 15-F2t-Isop was assessed with the ELISA Kit from Oxford Biomedical. Samples were reconstituted in 300 µL of dilution buffer (provided by the manufacturer) and analyzed following the manufacturer’s instructions.

Total antioxidant power (TAP) was assessed using the colorimetric kit provided by Oxford Biomedical Research (TA02 kit, Oxford Biomedical Research, Rochester Hills, MI, USA). All procedures were performed according to the manufacturer’s instructions.

### 2.3. Statistical Analysis

Statistical analysis was performed using SPSS (version 30.0) and Jamovi software (version 2.6.26). Biological data are presented as means (±standard deviations) and medians (IQRs), while categorical variables are presented as frequencies, using the number of observations. Due to the low number of observations (<30) and the non-Gaussian distribution of the biological data collected as ascertained by the Lavene test, non-parametric tests were used to assess differences and paired comparisons among and within groups (Mann–Whitney and Wilcoxon tests). A two-tailed *p*-value of less than 0.05 was considered indicative of statistical significance.

## 3. Results

Table 1 shows the concentration of 15-F2t-Isop quantified in pasteurized and non-pasteurized milk originating from the six donors. Table 1 also shows the same quantification categorized by sampling day. Despite the limited number of observations, the differences are statistically significant when considering both sampling campaigns together (12 observations) and separately (6 observations). The 15-F2t-Isop content in HoP DM milk was significantly lower (*p* < 0.001) if compared to non-HoP DM concentrations (0.8 ng/mL IQR 0.6–1.5 vs. 3.2 ng/mL IQR 1.6–5.3), suggesting potential degradation of this biomarker due to the pasteurization process.

**Figure 1:**
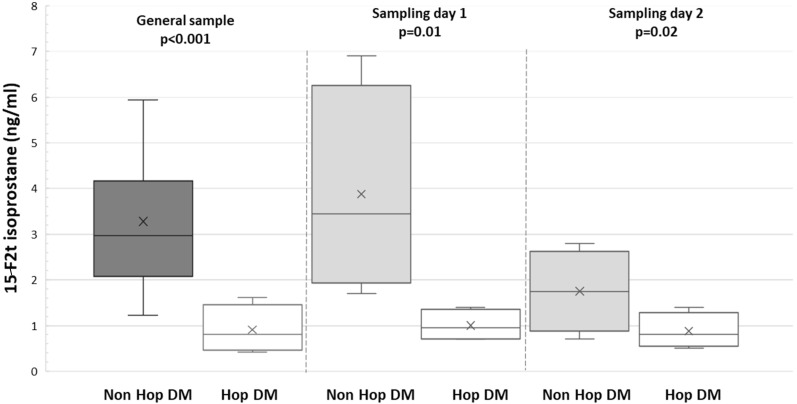
15-F2t-Isop in non-pasteurized and pasteurized (HoP) DM. All values are expressed as ng/mL. Darker grey indicates comparisons in the overall sample, whereas lighter grey indicates comparisons within the different sampling days.

This finding is corroborated by data stratified by sampling day (Table 1), in which the 15-F2t-Isop concentration in HoP DM was significantly lower when compared to non-HoP DM; Day 1: 1.0 ng/mL (0.5–1.7) vs. 3.5 ng/mL (2.6–6.5), *p* < 0.01; Day 2: 0.7 ng/mL (0.6–1.4) vs. 2.2 ng/mL (1.4–4.3), *p* < 0.02). This supports the hypothesis that the HoP process determines the degradation of 15-F2t-Isop. Figure 1 provides a graphical representation of the difference in 15-F2t-Isop quantities between pasteurized and non-pasteurized DM.

Regarding results for total antioxidant power, no significant results were identified between these groups (Table 2).

## 4. Discussion

Human milk is a complex biological matrix characterized by a diverse composition that plays a crucial role in immune and neurodevelopment of newborn babies [50,51,52]. Human milk is indeed a rich source of nutrients and bioactive compounds, including antioxidants that are readily transferred from mother to infant and contribute to protection against free radicals. When own mother’s milk is unavailable, particularly for preterm infants, donor human milk represents a fundamental alternative. Holder pasteurization is the most widely adopted method to ensure microbiological safety in milk banks; however, its biological impact on redox-related components remains incompletely understood and warrants further investigation [53]. OS is a central mechanism in the pathogenesis of several neonatal conditions, including bronchopulmonary dysplasia, retinopathy of prematurity, necrotizing enterocolitis, and periventricular leukomalacia [5,54,55]. For this reason, the preservation of antioxidant defense in DM is crucial from a clinical point of view [5]. In this regard, it is essential to verify that the positive nutritional properties of DM are not modified by conservation treatments such as refrigeration or pasteurization [56]. The 15-F2t-isoprostane represents a reliable biomarker of lipid peroxidation and oxidative damage in biological matrices. Previous studies have primarily investigated its stability during refrigeration and storage, reporting no significant degradation over time, results highlighted by Peila et al. [49]. To further investigate the effects of preservation treatments on milk, this study focused on the outcomes of the HoP process, which, in part, still need to be elucidated. Our results demonstrated a significant reduction in the 15-F2t-Isop content of DM samples subjected to the HoP process. This result is important from a methodological point of view because, for future studies, it needs to be kept in mind that a reduction in 15-F2t-Isop content can occur after pasteurization, likely due to the temperature reached during the process [57]. Therefore, the HoP process may reduce the level of OS transferred from the mother to the newborn [54,58,59], producing a beneficial effect, as postpartum is characterized by higher levels of OS [55], which can pass to milk and to the baby. Although this finding may suggest a lower oxidative burden transferred to the newborn, it cannot be unequivocally interpreted as a true improvement in redox balance. We explicitly considered thermal exposure during pasteurization as a plausible mechanistic explanation for this reduction, potentially through chemical modification or partial degradation of the biomarker. This remains one of the most likely hypotheses underlying the observed decrease. Consequently, the apparent reduction in oxidative damage could be interpreted as a processing-related effect rather than a definitive biological benefit. Moreover, this observation should not be interpreted as implying a nutritional superiority of HoP-treated milk. The primary objective in donor milk processing remains the preservation of a nutritionally rich and biologically valuable product capable of supporting optimal neonatal growth and development. Regarding the antioxidant capacity of human milk, our findings show that pasteurization did not appear to significantly affect the overall antioxidant potential. These results contrast with much of the existing literature, in which a reduction in antioxidant capacity after pasteurization is commonly reported. Such reductions are generally attributed to the heat sensitivity of enzymatic and non-enzymatic antioxidant components involved in reactive oxygen species scavenging. In particular, previous studies by [5,60], as well as the review by [61], have highlighted a detrimental effect of pasteurization on the antioxidant properties of human milk. The discrepancy between our findings and previous studies strongly suggests that the colorimetric methodology applied in the present work may not be fully suitable for this complex biological matrix. Matrix interference effects and limitations in sample preparation procedures may have compromised accurate quantification of antioxidant potential in human milk. Therefore, rather than indicating a true absence of pasteurization effects, our TAP results likely reflect methodological constraints of the current analytical approach. Future studies should focus on optimizing extraction protocols and analytical conditions to enable reliable measurement of antioxidant capacity in milk. Moreover, there is concern that the pasteurization process may also decrease certain beneficial nutritional components in DM, resulting in a lower availability of these elements for the newborn [36,49]. Common non-invasive matrices like urine can be influenced by the altered inflammatory profile of the mother following the multiple changes that occur during pregnancy and a traumatic event like delivery [62]. On the other hand, the analysis of milk does not need any adjustment in evaluating metabolites, unlike urine, in which renal function needs to be considered [63]. Finally, the ELISA-based quantification of 15-F2t-isoprostane demonstrated good feasibility as a cost-effective alternative to more technically demanding techniques such as liquid chromatography–mass spectrometry (LC-MS). Together with refined antioxidant assays, these approaches may facilitate larger-scale and epidemiological investigations of oxidative stress in human milk.

The main limitation of our study was the low number of samples collected and analyzed. Another limitation is related to the use of a colorimetric assay for milk analysis to evaluate the TAP concentration. Despite sample preparation being performed according to the manufacturer’s instructions, the milk matrix remained turbid, which may have interfered with optical measurements and potentially limited the accuracy of the assay. Among the strengths of this study, we can list (a) a standardized sample collection method, established by the HMB, which guarantees sample quality and reproducibility, (b) the use of an ELISA kit and a colorimetric kit, both already validated using urine [40,41,54], which guarantees reproducibility of the method and comparability of results for future studies on multiple matrices, and (c) the use of an ELISA kit, which is easier, cheaper, and quicker when compared to the other techniques. Using an ELISA kit is valuable, especially for epidemiological studies in which a large number of subjects are enrolled and for which it would therefore be too expensive to use the analytical gold standard for the measurement of content in milk.

Considering this, and following the conclusion of a previous study that has already investigated this point [64], a possible future study could test both the newborn’s urine and the mother’s milk, using the same kit to identify potential relationships between the mother’s OS level and the baby’s antioxidant capacity.

## 5. Conclusions

To our knowledge, this is the first study to evaluate the concentration of 15-F2t-isoprostanes in human milk using an ELISA assay, together with the assessment of total antioxidant power by a colorimetric method. Our positive result for the quantification of 15-F2t-isoprostanes will allow us to quantify this analyte in milk matrices with the same method.

This study provides evidence of the extensive degradation of 15-F2t-Isop following the HoP process in DM. This information should be considered in future research examining the OS transmitted from mothers to their own newborns and as milk donors. In conclusion, the reduction in 15-F2t-isoprostane levels observed following the HoP process may be considered favorable from the perspective of oxidative stress exposure in premature infants who rely on donor milk. However, this potential benefit should always be interpreted in the broader context of pasteurization-related effects on the nutritional and bioactive quality of human milk, which remains a critical determinant of neonatal health.

## Figures and Tables

**Table 1:** 15-F2t-Isop in pasteurized (HoP) and non-pasteurized DM, including samples subgrouped by sampling day. All values are expressed as ng/mL.

15 F2t Isoprostane (ng/mL)									
**Donor Milk**				**HoP Milk**					
**Mean**	**SD**	**Median**	**IQR**	**Mean**	**SD**	**Median**	**IQR**	**Mann–Whitney** ***p*** **Value**	**Wilcoxon (Paired)** ***p*** **Value**
**3.58**	±2.36	3.16	1.59–5.27	1.01	±0.6	0.76	0.62–1.54	<0.001	
***Sampling day 1***									
**Donor Milk (*n* = 6)**				**HoP Milk (*n* = 6)**					
**Mean**	**SD**	**Median**	**IQR**	**Mean**	**SD**	**Median**	**IQR**		
**4.31**	±2.59	3.53	2.55–6.53	1.2	±0.7	0.99	0.51–1.74	0.01	0.03
***Sampling day 2***									
**Donor Milk (*n* = 6)**				**HoP Milk (*n* = 6)**					
**Mean**	**SD**	**Median**	**IQR**	**Mean**	**SD**	**Median**	**IQR**		
**2.85**	±2.05	2.22	1.36–4.34	0.93	±0.55	0.75	0.60–1.44	0.02	0.04

**Table 2:** TAP in DM and pasteurized (HoP) DM expressed as mmol Trolox equivalents.

TAP (mmol Trolox Equivalents)								
Donor Milk				HoP Milk				
Mean	SD	Median	IQR	Mean	SD	Median	IQR	Mann–Whitney *p* Value
**0.27**	±0.12	0.31	0.38	0.24	±0.07	0.24	0.23	0.37

## Data Availability

Data presented in this study are available on request from the corresponding author due to the samples belonging to the Human Milk Bank of Sant’Anna Hospital in Turin, Italy.
